# Using Smart Wearables to Monitor Cardiac Ejection ^†^

**DOI:** 10.3390/ecsa-5-05744

**Published:** 2018-11-14

**Authors:** Aristide Mathieu, Peter H. Charlton, Jordi Alastruey

**Affiliations:** Department of Biomedical Engineering, School of Biomedical Engineering and Imaging Sciences, King’s College London, King’s Health Partners, St Thomas’ Hospital, London SE1 7EH, UK

**Keywords:** wearable sensors, pulse wave, left ventricular ejection time, contractility, photoplethysmogram

## Abstract

An individual’s cardiovascular state is a crucial aspect of a healthy life. However, it is not routinely assessed outside the clinical setting. Smart wearables use photoplethysmography (PPG) to monitor the arterial pulse wave (PW) and estimate heart rate. The PPG PW is strongly influenced by the ejection of blood from the heart, providing an opportunity to monitor cardiac parameters using smart wearables. The aim of this study was to investigate the feasibility of monitoring left ventricular ejection time (LVET) and left ventricular contractility (LVC) from the PPG PW at the wrist. PPG PWs were simulated under a range of cardiovascular conditions using a numerical model of PW propagation. LVET and LVC were estimated from the first and second derivatives of the PPG PWs and compared to reference values extracted from the blood pressure PW at the aortic root. There was strong agreement between the estimated and reference values of LVET, indicating that it may be feasible to assess LVET from PPG signals, including those acquired by smart watches. The correlations between the estimated and reference values of LVC were less strong, indicating that further work is required to assess contractility robustly using smart wearables. This study demonstrated the feasibility of assessing LVET using smart wearables that could allow individuals to monitor their cardiovascular state on a daily basis.

## 1 Introduction

Wearable sensors routinely acquire a photoplethysmogram (PPG) signal, which is a measure of the arterial pulse wave (PW) [1]. Fitness bands and smart watches, such as the Samsung Gear, Apple Watch and Fitbit Charge devices, use the PPG for heart rate monitoring [2]. In addition, research is being conducted into estimating arterial blood oxygen saturation from PPG signals acquired by smart wearables, in the same manner as clinical pulse oximeters [3]. However, the PPG PW contains a wealth of additional information on the state of the heart which is not currently exploited.

**Figure 1:**
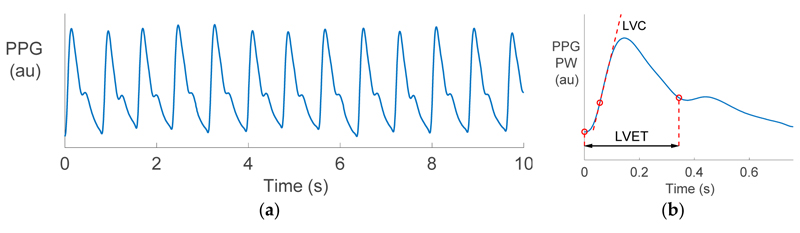
The photoplethysmogram (PPG) signal (**a**) A 10 s *in vivo* recording of the PPG, exhibiting 12.5 pulse waves (PWs), one for each heart beat. (**b**) The techniques for estimating left ventricular ejection time (LVET) and left ventricular contractility (LVC) from a single PW. au: Arbitrary units. Data from the Vortal dataset [12].

Photoplethysmography is the optical measurement of arterial blood volume changes in a tissue bed. Smart wearables usually obtain the PPG at the wrist by illuminating the skin with an LED and measuring the level of reflected light. A pulsatile signal consisting of PWs is produced as the arterial blood volume increases and decreases with each heart beat, as shown in Figure 1a. The PPG PW, shown in Figure 1b, has been found to be closely related to the arterial blood pressure (BP) PW, and the two signals have been related by a transfer function [4]. Consequently, it has been proposed that the methods used to estimate cardiac properties from the BP signal could be adapted for use with the PPG signal. However, there are challenges in extracting information from a PPG, including: (1) The shape of the PW changes as it propagates from the heart to the periphery, meaning that information may be distorted or lost. (2) High-frequency content is attenuated in the PPG, making it more difficult to identify features on the PPG PW than the BP PW. In this study we focus on estimating two cardiac parameters from the PPG: Left ventricular ejection time (LVET) and left ventricular contractility (LVC).

LVET is the time period of blood flowing through the aortic valve. LVET is influenced by several cardiac factors, including heart rate, stroke volume, preload, afterload and inotropic drugs [5]. Consequently, LVET is a valuable clinical parameter when monitoring the effects of drugs and diseases [5]. Techniques have been previously proposed for estimating LVET from BP [6–8] and PPG [9–11] PWs. LVET estimates derived from the BP PW have been found to perform well, whilst those derived from the PPG were found to be less accurate but still useful for detecting intra-subject changes in LVET [9]. If LVET could be reliably estimated from smart wearables then it would have utility for monitoring both health and fitness. However, at present it is not clear whether LVET could be accurately estimated from the peripheral PPG signals acquired by smart wearables, or whether accuracy would be improved by measuring BP signals in smart wearables.

Left ventricular contractility (LVC) is the strength of the contractile force generated by the heart [13]. Whilst it is difficult to assess *in vivo*, it can be quantified as the maximal rate of intraventricular pressure rise during systole [14]. Drugs can affect LVC, and consequently measurements of LVC can be valuable for assessing the safety of new drugs. It has been proposed that LVC could be assessed from the carotid BP signal [15]. However, to our knowledge no research has investigated whether LVC could be accurately assessed from PPG signals acquired by smart wearables.

The aim of this pilot study was to investigate whether LVET and LVC could be accurately estimated from peripheral PPG signals. Simultaneous BP and PPG PWs were simulated at the aortic root, wrist and carotid artery using a numerical model of pulse wave propagation. LVET and LVC values were estimated from PPG and BP PWs at the wrist and carotid artery, and compared to reference values obtained from the aortic root.

## 2 Materials and Methods

### 2.1 Dataset

The dataset used in this study was adapted from the database of simulated PWs presented in [16], which is publicly available [17]. This database was generated by simulating PWs using a numerical model of pulse wave propagation. The cardiac and arterial model parameters were varied within healthy ranges to produce PWs representative of 3,325 virtual healthy adult subjects. BP PWs at the aortic root and radial, brachial and carotid arteries were extracted from the database for this study. PPG PWs were estimated from the BP PWs using the transfer function reported in Reference [4] and implemented in [18]. PPG estimates were made at the wrist, representing those which would be measured from smart watches and fitness bands, and at the carotid artery, representing those which would be measured when using a smartphone camera on the surface of the neck to capture PWs.

### 2.2 Estimating LVET and LVC from Radial BP and PPG Waves

LVET and LVC were estimated from each of the radial BP and PPG waves using similar pulse wave analysis techniques to those reported in Reference [18]. The techniques are illustrated in Figure 1b. LVET was calculated as the time between the pulse onset and the “e” wave on the second derivative of the PW, since this is indicative of the time of aortic valve closure at the end of cardiac ejection [19]. This was performed by firstly filtering PWs to eliminate non-physiological, high-frequency content (using a low pass filter with a −3 dB cut-off at 35 Hz). Secondly, the first and second derivatives of PWs were calculated using a Savitsky–Golay filter. Thirdly, the “e” wave was identified as the maximum of the second derivative in a search region bounded by the systolic peak and 60% of the PW duration. LVC was calculated as the maximum value of the first derivative of the PW. For the BP PW it was measured in units of mmHg/s. For the PPG PW it was measured in au/s, where au represented arbitrary units (since the PPG is unitless).

### 2.3 Statistical Analysis

A fixed reference value for LVET of 310 ms was used, as this was not varied in the original database [16]. Errors between estimated and reference LVET values were calculated by subtracting the reference from estimated values. Reference values for LVC were obtained from aortic BP PWs using the same approach as for radial BP waves. The strength of the correlation between estimated and reference LVCs was assessed using the coefficient of determination R^2^, the square of Pearson’s correlation coefficient. Results were compared between PPG and BP PWs to determine whether it could be beneficial to include BP PW monitoring more widely in smart wearables.

## 3 Results and Discussion

### 3.1 Comparing PPG and BP Waves

**Figure 2:**
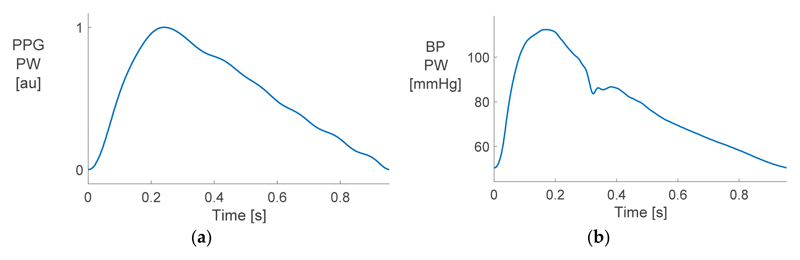
A comparison of (**a**) photoplethysmogram (PPG) and (**b**) blood pressure (BP) pulse waves.

Figure 2 shows examples of the PPG and BP PWs used in the analysis. The two PWs had different shapes, indicating that pulse wave analysis techniques which have been originally used with the BP PW may not be suitable for use with the PPG. Specifically, the BP PW contained more high-frequency features, such as a dicrotic notch on the downslope. These features were largely attenuated in the PPG PW. Consequently, it may be more difficult to estimate LVET from a PPG, as it relies on precise identification of the end of systole, which is indicated by the dicrotic notch.

### 3.2 Estimating Left Ventricular Ejection Time (LVET)

**Figure 3:**
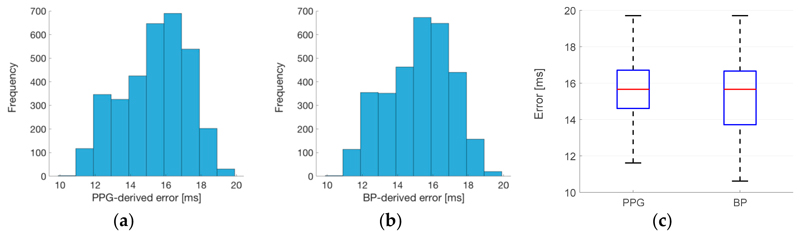
Estimating left ventricular ejection time (LVET) at the wrist (**a**) errors when using the photoplethysmogram (PPG) pulse wave (PW); (**b**) errors when using the blood pressure PW; and (**c**) a comparison of the errors when using each signal (showing median and lower and upper quartiles).

The results of estimating LVET at the wrist from the PPG and BP PWs are shown in Figure 3. The estimated LVETs were higher than the reference values, as shown by the positive errors in Figure 3a,b. If this bias was found to be consistent in future studies, then one could obtain more accurate estimates by simply subtracting the bias from estimated values. The errors ranged between 10 and 20 ms (i.e., 3% and 6%). Since typical values for LVET are approximately 295 ± 24 ms [6], these errors may be sufficiently small to allow for inter-subject comparisons and subject-specific risk stratification. There was little difference between the errors obtained when using the PPG and BP PWs, as shown in Figure 3c. This indicates that there would be no advantage to using the BP PW compared to the PPG PW. Therefore, the existing PPG hardware in smart wearables may be sufficient for obtaining clinically useful values of LVET.

### 3.3 Estimating Left Ventricular Contractility (LVC)

**Figure 4:**
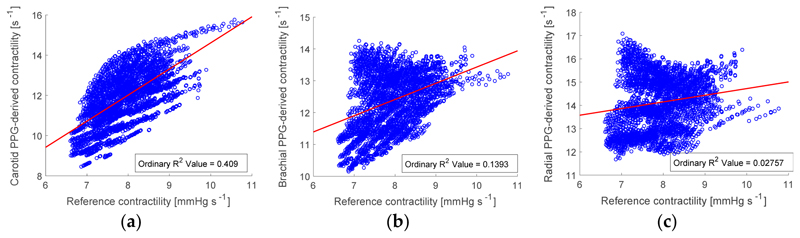
Estimating left ventricular contractility (LVC) from the PPG: the correlation between estimated and reference values at (**a**) the carotid artery (i.e., neck); (**b**) the brachial artery (i.e., upper arm); and (**c**) the radial artery (i.e., wrist). Least-squares lines are shown in red.

The results of estimating LVC from the PPG at the wrist are shown in Figure 4c, and for comparison results are also shown for the carotid artery (Figure 4a) and brachial artery (Figure 4b). The correlation of PPG-derived LVC values at the wrist was too low to be clinically useful (R^2^ = 0.03). This indicates that further work is required to obtain useful assessments of LVC at the wrist. However, the strength of the correlation was higher at arterial sites closer to the heart (carotid and brachial sites). In particular, the correlation of R^2^ = 0.41 at the carotid artery may be strong enough to provide useful assessments of LVC. Such assessments could potentially be performed using an imaging PPG signal acquired using a smartphone at the neck.

### 3.4 Limitations and Future Work

The main limitation of this pilot study was that it used simulated signals. This had the benefits of ensuring that reference parameter values were known precisely, without measurement error, and allowing the performance of techniques for estimating LVET and LVC to be assessed across a wide range of cardiovascular conditions. A second limitation was the use of a fixed value for LVET. The study indicated that further work is required to develop techniques to estimate LVC. In addition, the PPG signals routinely acquired by wearable sensors may be sufficient to assess LVET, without the need to acquire BP signals, which are more difficult to acquire from wearables.

Future studies should assess the performance of the technique for estimating LVET from smart wearables *in vivo*. Initial validation should be performed in controlled laboratory settings, where it is easier to acquire reference measurements. The technique could then be assessed in daily living, where less-precise reference measurements could be acquired to validate the technique. If validated, then this approach would allow LVET to be continuously monitored in daily life, improving health monitoring. It would also allow for large-scale research studies to be conducted into the utility of LVET for clinical decision making and diagnosis in various types of cardiovascular diseases, including aortic valve disease, left ventricular failure, stenosis and aortic regurgitation [20].

### 3.5 Implications

The inclusion of PPG sensors in smart wearables provides opportunity to monitor cardiovascular states unobtrusively in daily life. Photoplethysmography was first reported in the 1930s [21], and began being widely used in clinical practice in the 1980s with the proliferation of pulse oximeters for measuring arterial blood oxygen saturation [22]. The ability to unobtrusively measure oxygen saturation was deemed so revolutionary that it became considered the fifth vital sign [23]. Despite its routine use in clinical practice, the full utility of the PPG signal has not yet been exploited. A wide range of applications have been investigated in the research setting, including assessing arterial stiffness, respiratory rate, microcirculation, venous function and blood pressure [24], as well as the possibility of assessing LVET and LVC, which has been investigated in this study. It may be that the widespread use of PPG sensors in smart wearables provides sufficient incentive to translate more of these potential applications into routine practice.

## 4 Conclusions

The main finding of this pilot study was that LVET could be estimated from PPG signals at the wrist with sufficient precision for use in some settings. Clinical studies are warranted to determine whether this approach could be used to estimate LVET precisely from wrist-worn smart wearables. LVC could not be estimated precisely from the wrist using the trialed technique. Further development of pulse wave analysis techniques may improve the performance of LVC estimates, although this study indicates that they are likely to perform better when obtained from signals acquired at the neck, such as imaging PPG signals.
